# Repair of large segmental bone defects: BMP-2 gene activated muscle grafts vs. autologous bone grafting

**DOI:** 10.1186/1472-6750-13-65

**Published:** 2013-08-08

**Authors:** Oliver B Betz, Volker M Betz, Christian Schröder, Rainer Penzkofer, Michael Göttlinger, Susanne Mayer-Wagner, Peter Augat, Volkmar Jansson, Peter E Müller

**Affiliations:** 1 Department of Orthopedic Surgery, Laboratory for Biomechanics and Experimental Orthopedics, University Hospital Grosshadern, Ludwig-Maximilians-University Munich, Marchioninistrasse 23, Munich, 81377, Germany; 2 Institute of Biomechanics, Trauma Center Murnau, Prof.-Küntscher-Str. 8, Murnau, 82418, Germany

**Keywords:** Bone regeneration, Large bone defects, Muscle grafts, Gene transfer, BMP-2, Autologous bone grafts, *In vivo*, Tissue engineering

## Abstract

**Background:**

Common cell based strategies for the treatment of osseous defects require the isolation and expansion of autologous cells. Since this makes such approaches time-consuming and expensive, we developed a novel expedited technology creating gene activated muscle grafts. We have previously shown that large segmental bone defects in rats can be regenerated by implantation of muscle tissue fragments activated by BMP-2 gene transfer.

**Results:**

In the present study, we compared the bone healing capacities of such gene activated muscle grafts with bone isografts, mimicking autologous bone grafting, the clinical gold standard for treatment of bone defects in patients. Two of 14 male, syngeneic Fischer 344 rats used for this experiment served as donors for muscle and bone. Muscle tissue was harvested from both hind limbs and incubated with an adenoviral vector carrying the cDNA encoding BMP-2. Bone was harvested from the iliac crest and long bone epiphyses. Bone defects (5 mm) were created in the right femora of 12 rats and were filled with either BMP-2 activated muscle tissue or bone grafts. After eight weeks, femora were evaluated by radiographs, micro-computed tomography (μCT), and biomechanical testing. In the group receiving BMP-2 activated muscle grafts as well as in the bone-grafting group, 100% of the bone defects were healed, as documented by radiographs and μCT-imaging. Bone volume was similar in both groups and biomechanical stability of the two groups was statistically indistinguishable.

**Conclusions:**

This study demonstrates that treatment of large bone defects by implantation of BMP-2 gene activated muscle tissue leads to similar bone volume and stability as bone isografts, mimicking autologous bone grafting.

## Background

Large bone defects are hard to treat and cause pain, disability and high cost. Autologous bone grafting is the clinical gold standard for the regeneration of large defects in patients. However, the limited amount of bone tissue available for autografting and donor site morbidity represent significant drawbacks of this method [1-3]. Bone morphogenetic proteins are clinically used for the treatment of long-bone fractures and for enhanced spinal fusion [4-8] but their delivery needs improvement due to their short biological half-lives [9,10]. Gene transfer is an improved way of delivering such growth factors as it is possible to achieve high concentrations locally for an extended period of time [11-13]. A suitable vector for the purpose of inducing bone growth in a lesion is adenovirus as it is a non-integrating vector with high transduction efficiency. Additionally, *in vivo* expression stops after about six weeks which may just provide enough stimulus for bone repair avoiding the risk of excessive ectopic overgrowth of bone. In previous studies, we evaluated the effects of direct *in vivo* gene delivery to bone defects in a rat model [14-16] and generated encouraging data. However, to further improve bone repair by gene therapy, we believe that it is necessary to supply not only an osteogenic gene, but also stem cells and a scaffold. Most *ex vivo* gene therapy approaches utilize these three ingredients. The combination of growth factor over-expressing bone marrow derived cells [17,18], fat derived cells [19] and skin cells [20] together with a matrix has successfully induced bone formation *in vivo*. It has also been demonstrated that muscle derived stem cells have the potential to differentiate towards the osteogenic lineage and effectively regenerate bone defects in pre-clinical animal models [21,22]. Although *ex vivo* gene therapy has been very successful in pre-clinical bone repair models, the method is time-consuming and expensive as it requires the isolation and expansion of autologous progenitor cells. For this reason, we developed an expedited *ex vivo* gene therapy approach which is characterized by the direct transfer of BMP-2 cDNA to muscle tissue fragments without extracting cells from this tissue. In a recent study, we demonstrated that such expedited gene activated muscle grafts successfully induce repair of large segmental bone defects in rats [23]. The harvest of muscle tissue is a frequently used procedure in reconstructive surgery and only minor donor site morbidity was reported after harvesting the vastus lateralis muscle or the gracilis muscle [24,25]. Muscle tissue could therefore be a practicable source for the augmentation of bone defects after BMP-2 gene activation. Since autologous bone grafting is the clinical gold standard for the repair of osseous defects in patients we compared our novel expedited *ex vivo* gene therapy approach to this gold standard evaluating repair of critical-sized femoral bone defects in rats.

## Results

Six femora of the group treated with Ad.BMP-2 gene activated muscle grafts and six of the bone grafting group were evaluated by radiographs, micro-computed tomography (μCT) and biomechanical torsional testing. Intra- and postoperatively, there were no complications and we did not see wound infections or delayed wound healing. The rats were able to bear their full weight on the operated limbs from the day of surgery until they were euthanized. Gene activated muscle grafts were well tolerated and produced no obvious adverse effects during the eight weeks of the experiment. All animals showed a normal eating pattern and no weight loss was recorded.

### Measurement of BMP-2 up-regulation by ELISA

On day six after transduction BMP-2 up-regulation was confirmed by ELISA. BMP-2 transduced muscle discs produced 5260 ± 3158 pg/ml BMP-2. BMP-2 concentration in supernatants of controls was 68 ± 52 pg/ml (unmodified muscle).

### Radiographic evaluation

**Figure 1:**
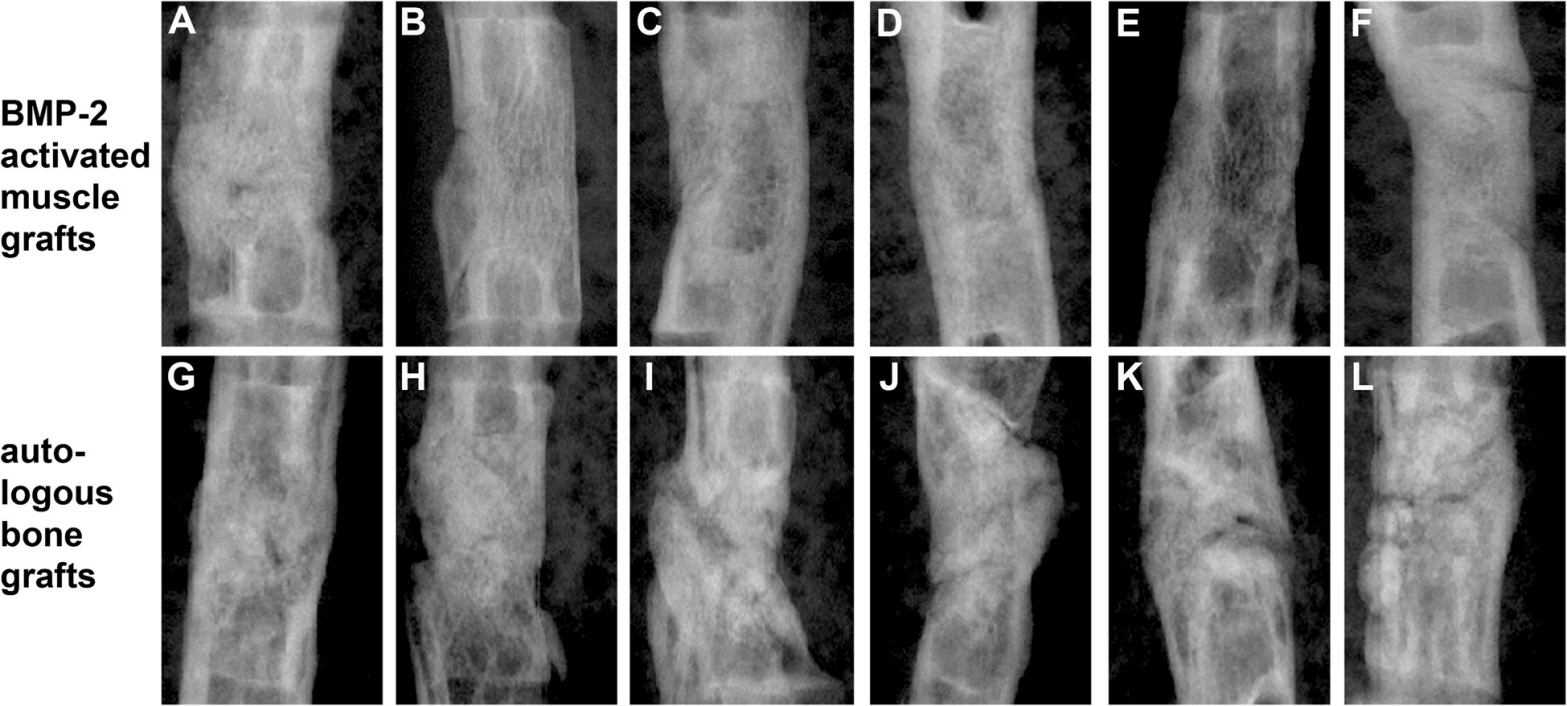
**Radiographic images.** Radiographic images of femoral segmental bone defects 8 weeks after surgery. Femora treated with BMP-2 gene activated muscle grafts **(A **-** F)** and autologous bone grafts **(G** – **L)** displayed complete bridging of the defect.

100% (six out of six) of the animals treated with Ad.BMP-2 gene activated muscle grafts showed united bones at eight weeks after treatment (Figure 1A-F), and 100% (six out of six) of the femora treated with bone grafts appeared united radiographically (Figure 1G-L).

### Micro-computed tomography (μCT)

**Figure 2:**
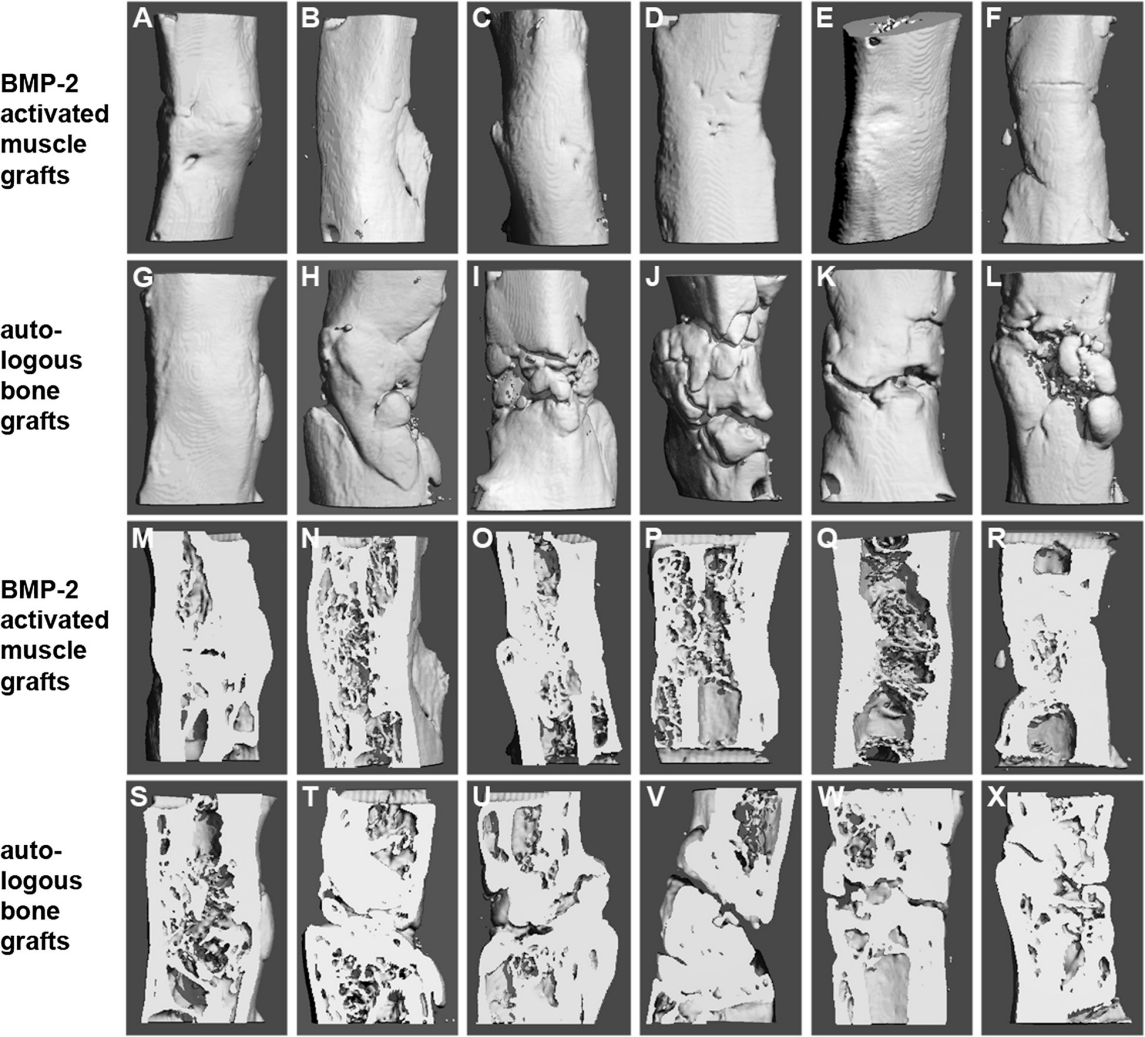
**Micro-CT images.** Micro-CT images of femoral segmental bone defects treated with BMP-2 gene activated muscle grafts **(A** – **F)** and autologous bone grafts **(G **– **L)** and longitudinal, medial sections of micro-CT images of femoral segmental bone defects treated with BMP-2 gene activated muscle grafts **(M** – **R)** and autologous bone grafts **(S** – **X)** 8 weeks after surgery. All images of the femora treated with BMP-2 gene activated muscle grafts **(A **– **F** and **M** – **R)** displayed complete bridging and regeneration of cortical bone and cancellous bone formation in the medullary cavity. The micro-CT-images of the autologous bone graft group **(G** – **L** and **S** – **X)** revealed incomplete bridging of several defects **(U**, **V **and **W)**.

*Images:* All six femora receiving Ad.BMP-2 gene activated muscle grafts displayed bridging of the 5 mm defects at eight weeks (Figure 2A-F and M-R). In the bone grafting group, *μCT*-imaging, in contrast to the conventional radiographic evaluation (Figure 1), reveals incomplete bridging of some defects (Figure 2G-L and S-X).

#### Bone volume

**Figure 3:**
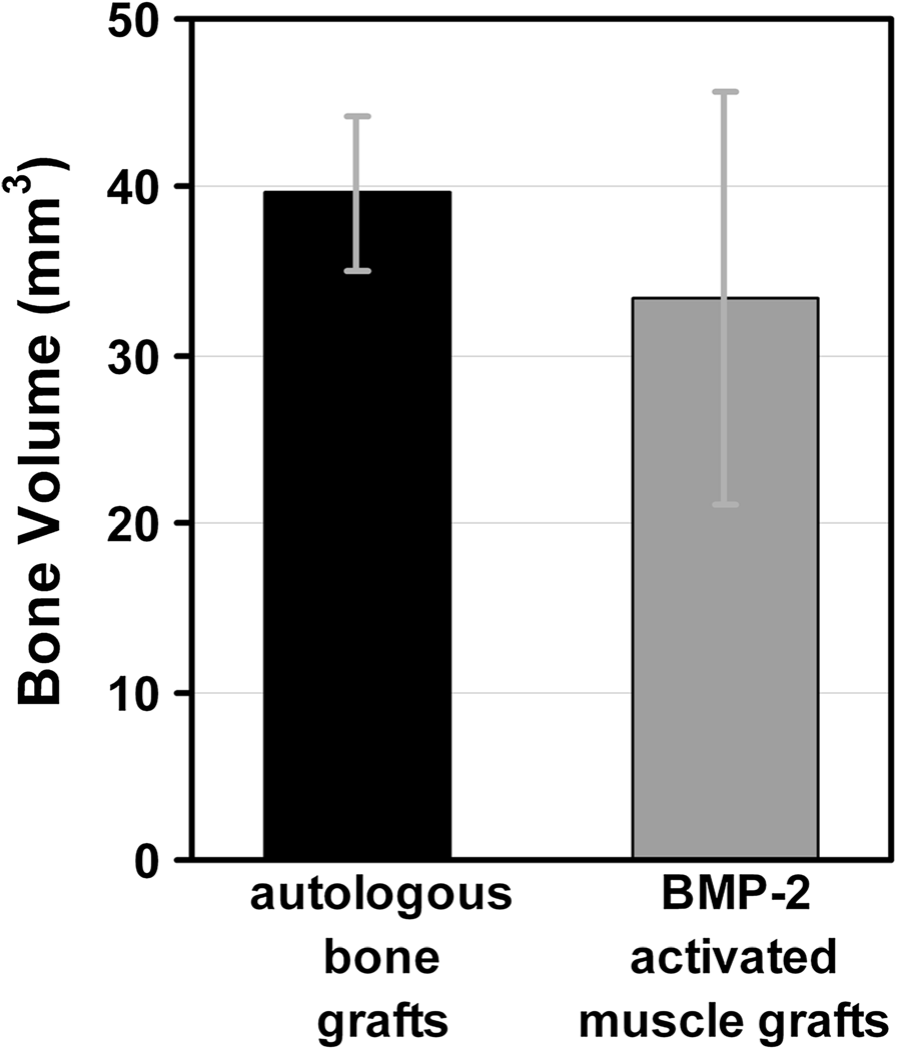
**Bone volume determined by micro-CT.** Micro-CT evaluation of the bone volume of defects treated with autologous bone grafts and BMP-2 activated muscle grafts. Values given represent means ± SD; (autologous bone grafts, n = 6; BMP-2 activated muscle grafts, n = 6). No statistically significant difference in bone volume of femora receiving autologous bone grafts versus BMP-2 activated muscle grafts could be determined (p = 0.12).

The bone volume of femora treated with BMP-2 gene activated muscle tissue (33.44 ± 12.26 mm^3^) was statistically indistinguishable from that of femora treated with bone grafts (39.66 ± 4.63 mm^3^) (p = 0.12) (Figure 3).

### Biomechanical testing

**Figure 4:**
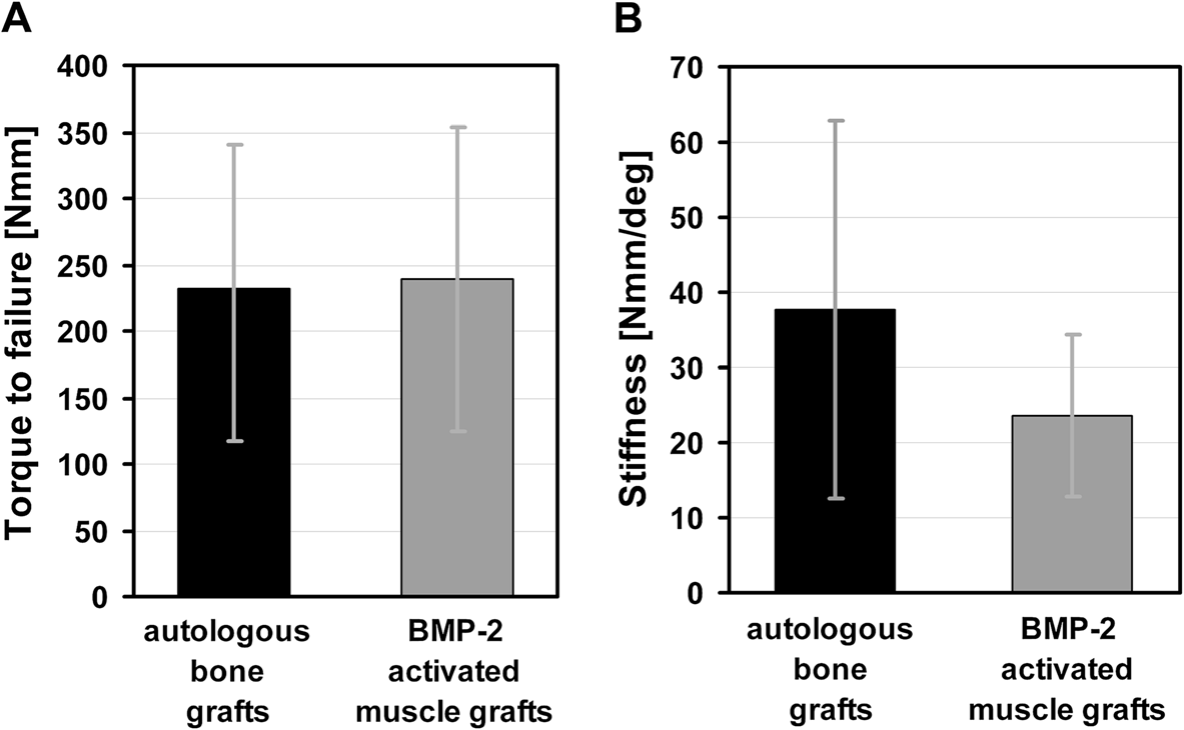
**Mechanical properties of bones. (A)** Torque to failure and **(B)** stiffness of femora treated with autologous bone grafts and BMP-2 activated muscle grafts 8 weeks after surgery. Values given represent means ± SD; (autologous bone grafts, n = 6; BMP-2 activated muscle grafts, n = 6). No statistically significant difference in torque to failure (p = 0.87) or stiffness of femora receiving autologous bone grafts versus BMP-2 activated muscle grafts could be determined (p = 0.42).

The mechanical properties of the femora treated with BMP-2 activated muscle grafts were evaluated by torsional testing and compared to properties of femora treated with bone grafts. Femora treated with BMP-2 activated muscle grafts showed a torque to failure (238.47 ± 114.54 Nmm) and a torsional stiffness (23.50 ± 10.79 Nmm/rad) that were in the range of the femora treated with bone grafts (231.59 ± 108.36 Nmm) respectively (37.70 ± 25.03 Nmm/rad) with no statistically significant difference (p = 0.87 and p = 0.42, respectively) (Figure 4-A and 4-B).

## Discussion

The rare disease *fibrodysplasia ossificans progressiva (FOP)* taught us that constitutive activation of a BMP type I receptor causes bone growth in muscle tissue [26]. The fact that BMP signaling leads to bone formation in muscle was exploited in our experiment and enabled us to develop a novel abbreviated *ex vivo* gene transfer approach to bone healing. In this study, bone formation was reliably induced after implantation of BMP-2 activated muscle tissue and all defects of this group were bridged at eight weeks. Bone volumes measured in former defect areas were similar after treatment with gene activated muscle compared to autologous bone grafting. Biomechanical stability was statistically indistinguishable. We repeatedly report successful bone defect repair using the gene activated muscle graft technology. In addition to our previous study where we evaluated healing in the same animal model [23], we were now able to demonstrate that BMP-2 activated muscle repairs large bone defects as effectively as bone grafting. The reason for this success might be that we not only delivered BMP-2 in a sustained fashion but also provided a natural degradable scaffold and stem cells by implanting muscle tissue. To our knowledge, this is the first comparison between a cell-based bone repair technology and autologous bone grafting in a pre-clinical large segmental bone defect model. Many researchers may eschew this comparison but we believe that, right from the beginning in a small animal model, a novel approach must demonstrate at least equal effectiveness as compared to the clinical gold standard. The fact that we see similar effectiveness encourages us to further develop and improve the technology in order to exceed the efficacy of autologous bone grafting. Compared to bone autograft muscle tissue can be harvested in larger quantities causing only low donor site morbidity [24,25] and, once gene transfer to muscle grafts has been optimized, it might be a more potent graft material showing higher osteoinductivity leading to faster and more robust bone defect healing.

Soft tissue defects require the use of muscle flaps frequently. It was shown, that the harvest of the vastus lateralis or the gracilis muscle only leads to minimal donor site morbidity and even the complete absence of these muscles rarely caused lower extremity weakness [24,25]. The harvest of either of those muscles in humans should enable us to fill even large, critical size bone defects. Upscaling to a large animal model using autograft muscle tissue is the focus of our current research activity.

Conventional *ex vivo* approaches for bone regeneration have been successful in pre-clinical experiments but could not enter widespread clinical use due to complexity and high cost. The most expensive steps are cell isolation and expansion of autologous progenitor cells performed over several weeks under GMP conditions. Instead, if production of a cell-based implant took only 1–2 days chances for translation into a clinical setting rose. Another expedited gene transfer strategy is the direct percutaneous injection of viral vector into a bone lesion. This approach was investigated in our former studies and led to bone formation in the same femoral defect rat model. However, bone healing was not as robust and reliable as seen with the here presented muscle graft method. Also, the direct *in vivo* gene transfer led to ectopic bone formation due to leakage of the viral vector.

## Conclusion

We conclude that the here presented gene activated muscle graft technology can be considered as an expedited approach combining safety features of *ex vivo* gene therapy with the simplicity of a direct *in vivo* approach, especially when applied during a single surgical step. This novel abbreviated gene-enhanced bone repair method should have the potential to be performed during a single surgery as genetically manipulation by adenovirus can be completed within 1 to 2 hours. To demonstrate this, in one of our next studies we will take muscle tissue from a rat, genetically modify the tissue fragments intra-operatively and re-implant the gene activated tissue into a bone defect of the same rat. Expedited, cost-effective bone regeneration strategies are of great interest as economic burdens impede translation of most common cell-based therapies. In this rat study we were able to show that BMP-2 over-expressing autologous muscle grafts repair large segmental bone defects as effectively as autologous bone grafts.

## Methods

### Study design

14 male syngeneic Fischer 344 rats (weight 300–350 g) were used for this study. Bone and muscle tissue were harvested from two euthanized donor rats. A 5 mm, critical sized mid-femoral defect was created in the right hind limb of each of 12 rats and stabilized by an internal fixator. The rats were divided in two groups. Bone defects received either muscle grafts activated by an adenoviral vector carrying the human BMP-2 cDNA (Ad.BMP-2) (n = 6) or bone grafts (n = 6). At eight weeks after surgery healing of the femora was evaluated by radiographs, micro-computed tomography (μCT) and biomechanical testing.

### Vector production

Serotype 5, E1, E3 deleted, first generation adenoviral vector was constructed by amplification of the hBMP-2 coding region by PCR-amplified cloning it into the shuttle vector pO6-A5-CMV. Correctness of the hBMP-2 open reading frame in the resulting vector pO6A5-CMV-hBMP-2 was confirmed by sequence analysis. The CMV-BMP-2 portion of pO6A5-CMV-hBMP-2 was then transferred via recombination in a BAC vector containing the genome of a replication deficient Ad5-based vector deleted in E1/E3 genes. Following release of the recombinant viral DNA by restriction digest with PacI HEK-293 cells were transfected with the adenoviral DNA and infectious particles were generated. The amplified viral particles were chromatographically purified using ViraBind™ Adenovirus Purification Kit (Cell Biolabs Inc.). Infectious titers were determined via immunohistochemical detection of the adenoviral hexon protein in infected HEK 293 cells.

### Tissue harvest

Bone and muscle tissue were harvested from two donor animals. Bone was taken from the iliac crest and epiphyses of long bones and muscle was harvested from the upper thighs. Both tissues were washed with phosphate buffered saline and transferred into a Petri dish. Muscle tissue was cut under sterile conditions into slices of approximately 1 mm thickness. Discs were then punched out using a 4 mm dermal biopsy punch to create fragments of a standardized size and placed in 24-well plates. No cell isolation was performed. Muscle tissue was then transduced with an adenoviral BMP-2 vector (see “Activation of Muscle Tissue Grafts”). Harvested bone was cut into 1 to 2 mm fragments and placed in 24-well plates. DMEM media (BiochromeAG, Berlin, Germany) was added to the bone fragments and grafts were placed in an incubator for 24 – 48 hours prior to implantation. Fischer 344 rats are genetically identical animals that allow transplantation of tissue from one individual to another without inducing a host-versus-graft immune response known from allograft transplantation [27-29]. For practical and logistical reasons we used this well established syngeneic rodent autotransplant model, harvested bone and muscle tissue from two euthanized donor rats, implanted the tissue into femoral bone lesions of 12 syngeneic rats and thereby mimicked autograft transplantation.

### Activation of muscle tissue grafts

Right after harvest muscle tissue fragments were infected with 1 × 10^8^ plaque forming units (pfu) of Ad.BMP-2. Viral particles were appropriately diluted in phosphate buffered saline (PBS) and a volume of 10 μL was applied directly to the surface of the tissue grafts using a micro pipette. Then the tissue fragments were placed in an incubator (37°C, 5% CO_2_) for 60 minutes. In order to avoid dilution of the vector concentration no cell media was added to the tissue. The virus solution and the humidified air in the incubator kept the tissue fragments moist during incubation. Then DMEM media (BiochromeAG, Berlin, Germany) was added and the muscle tissues were placed back in the incubator for 24 – 48 hours prior to implantation.

### Measurement of BMP-2 up-regulation by ELISA

Four muscle discs in 700 μl of DMEM media per well were held in culture using a 24-well plate. Media was changed every third day. The supernatants of untreated muscle and BMP-2-transduced muscle were harvested on day six and BMP-2 up-regulation was measured by ELISA. Measurements were performed in quadruplicate (n = 4).

### Surgical procedure

All operative procedures were approved by the animal welfare committee of Bavaria, Germany (“Regierung von Oberbayern”). A modified version of an established, critical-size, femoral defect rat model [14,16,30], was used in this study. The animals were placed under general anesthesia by the administration of isoflurane using a small-animal vaporizer at initially 4% isoflurane in 2 L O_2_/min and then 2% isoflurane in 2 L O_2_/min to maintain. Following anesthesia, the animals received intramuscular injections of 0.025 mg/kg buprenorphine (analgetic) into the left thigh. After aseptical preparation for surgery, an approximately 40 mm long skin incision was created over the right femur and underlying soft tissue was retracted to reveal the bone. Two transcortical holes were drilled in a latero-medial plane in the proximal femur using a 0.9 mm drill bit (Gebr. Brasseler GmbH, Lemgo, Germany) and a second pair was placed in the distal femur, leaving enough space for creating the defect. Four pins (1.1 mm, threaded Kirschner-wires) (MicroAire, Charlottesville, VA, USA) were then placed into the femur and cut off to a length of approximately 20 mm. Two fixator plates were placed on top of each other and secured to the ends of the pins using mini-screws. A 5 mm osteotomy was then created in the center of the bone between the two inner pins using a sterile, round dental burr (Gebr. Brasseler GmbH, Lemgo, Germany) attached to a dental hand piece (Modell 174025, Eichenmayer KG, Tuttlingen, Germany). After completion of the osteotomy, the site was copiously irrigated with saline.

### Implantation of bone and muscle tissue grafts

Excessive vector was washed off by placing the muscle tissue grafts in a 50 ml Falcon tube filled with PBS. PBS was replaced four times and the tube was shaken gently. The muscle grafts were then implanted within the segmental bone defects of six animals. A staple of five tissue fragments was “press fitted” into the defect without securing the implants to the periosteum. Another six animals received bone grafts that were washed four times with PBS prior to implantation. After implantation of the grafts the muscle fasciae were closed using 3–0 chromic cat gut suture. The two fixator plates were lowered down consecutively to a distance of about 5 mm above the surrounding muscle surface and secured to the pins. The pins were cut to be flush with the plates and the skin incision was closed over the plates using 4–0 silk suture. Right after surgery, the animals received an intramuscular injection of 0.025 mg/kg buprenorphine for additional analgesia. During the first 48 hours after surgery, each animal received intramuscular injections of 0.05 mg/kg buprenorphine every 12 hours. Animals were killed 8 weeks after surgery. The femora were harvested, fresh frozen and stored at −20°C prior to evaluation by X-ray, micro-computed tomography and biomechanical testing.

### Radiographic evaluation and Micro-computed tomography (μCT)

Femora were scanned using a desktop micro-tomographic imaging system (μCT80, Scanco Medical AG, Brüttisellen, Switzerland) equipped with a 10 mm focal spot microfocus X-ray tube. The scout view X-ray image was saved for the radiographic evaluation. The entire defect region was scanned using a 20 μm isotropic voxel size, at 70 keV energy, 400 ms integration time and requiring approximately 420 μCT slices per specimen. In the defect region only 3 mm bone volume was analyzed for each specimen to ensure that no old cortical bone was included in the analyses. Images were thresholded using an adaptive-iterative algorithm [31,32] and morphometric variables were computed from the binarized images using direct, 3D techniques that do not rely on any prior assumptions about the underlying structure [33].

### Biomechanical testing

Following imaging, 6 femora treated with BMP-2 activated muscle grafts and 6 femora treated with bone grafts were tested to failure in torsion on a biaxial testing machine (Instron 8874; High Wycombe, UK). Both ends of each specimen were embedded using PMMA (Technovit 3040; Heraeus Kulzer, Germany) to provide an appropriate and reproducible gripping interface with the testing module. In order to avoid lateral forces and bending moments caused by clamping, a special manufactured device was used to place the specimen during the embedding process. This enabled us to embed the bone ends in the center of the PMMA disc and aligned the longitudinal axis of the bones with the torsion axis. Specimens were tested to failure rotation at a constant deformation rate of 5 rad/min angular deformation and applied load data were acquired at 10 Hz. Due to the potential shortening of the femora during torsion and the resulting tensile force along the longitudinal axis of the bones, a constant axial load of 5 N was applied during the test. The torsional stiffness was calculated from each torque rotation diagram. The torque to failure was defined as the first maximum in the torque rotation diagram.

### Statistical methods

Statistical significance of the biomechanical data and the bone volume data was determined by using the Mann–Whitney *U*-test. (p < 0.05 was considered statistically significant).

## Competing interests

The authors declare that they have no competing interests.

## Authors’ contributions

OB conceived the study, carried out animal surgery and participated in data interpretation and drafting the manuscript. VB made substantial contribution to the conception and design, carried out animal surgery and participated in data interpretation and drafting the manuscript. CS participated in biomechanical testing, participated in biomechanical data interpretation and drafting the manuscript. RP performed the biomechanical testing and participated in drafting the manuscript. MG performed the microCT evaluation, participated in biomechanical data interpretation and drafting the manuscript. SM performed the ELISA-measurement, data interpretation and drafting the manuscript. PA made substantial contribution to the conception and design of the biomechanical and micro CT evaluation, participated in biomechanical and microCT datainterpretation and drafting the manuscript. VJ contributed to the conception and design by adding the clinical insight and contributed to the biomechanical evaluation. PM participated in the design and the coordination of the study and helped to draft the manuscript. All authors read and approved the final manuscript.
